# Liposomal Myricetin Nanoantioxidants Attenuate Methotrexate-Induced Hepatotoxicity by Modulating Oxidative Stress, Inflammation, and Apoptosis in Rats

**DOI:** 10.3390/antiox15040452

**Published:** 2026-04-04

**Authors:** Fahad Alshammari, Ekramy M. Elmorsy, Abdulrahman S. Aldaghmi, Fahd Alaajam, Eida M. Alshammari, Mona M. Elghareeb, Manal S. Fawzy, Noha M. Abd El-Fadeal

**Affiliations:** 1 Department of Biology, College of Science, Jouf University, Sakaka 72341, Aljouf, Saudi Arabia; fahadm@ju.edu.sa (F.A.); asaldaghmi@ju.edu.sa (A.S.A.); 2 Center for Health Research, Northern Border University, Arar 73213, Saudi Arabia; ekramy.elmorsy@nbu.edu.sa; 3 Department of Medical Laboratory Technology, College of Nursing and Health Science, Jazan University, Jazan 45142, Saudi Arabia; falaajam@jazanu.edu.sa; 4 Department of Chemistry, College of Sciences, University of Ha’il, Ha’il 55473, Saudi Arabia; eida.alshammari@uoh.edu.sa; 5 Department of Physiology, Faculty of Veterinary Medicine, Mansoura University, Mansoura 35516, Egypt; monael_ghareeb@mans.edu.eg; 6 Department of Medical Biochemistry and Molecular Biology, Faculty of Medicine, Suez Canal University, Ismailia 41522, Egypt; noha_abdelfadeal@med.suez.edu.eg; 7 Department of Biochemistry, Ibn Sina National College for Medical Studies, Jeddah 22421, Saudi Arabia

**Keywords:** myricetin, liposomal nanoparticles, nanoantioxidants, methotrexate-induced hepatotoxicity, oxidative stress, inflammation, apoptosis, NRF2/HO-1 signaling, MAPK pathway

## Abstract

Methotrexate (MTX) is widely used for its chemotherapeutic and immunosuppressive properties, but is limited by oxidative stress-mediated hepatotoxicity. Nanoantioxidant delivery systems can enhance the stability, solubility, and in vivo efficacy of natural antioxidants. This study investigated the hepatoprotective effects of myricetin (MYR), a flavonoid with potent antioxidant activity, and its liposomal nanoantioxidant formulation (MYR-loaded liposomal nanoparticles, MYR-LNPs) against MTX-induced liver injury in male albino Sprague Dawley rats. Sixty rats were randomly allocated to six groups: control, MTX, MYR, MYR-LNPs, and combinations of MTX with MYR-LNPs. MYR-LNPs were successfully formulated and physicochemically characterized, exhibiting a mean particle size of 95.6 nm, a zeta potential of −32 mV, and a narrow polydispersity index, collectively confirming their colloidal stability and suitability for hepatic delivery. MTX markedly disrupted liver function, increasing serum AST, ALT, ALP, and bilirubin and decreasing total protein, albumin, and globulin, whereas co-treatment with MYR-LNPs substantially restored these parameters and outperformed free MYR. MTX-induced oxidative stress, reflected by depleted hepatic GSH and antioxidant enzymes (GPx, SOD, CAT, GST), elevated reactive oxygen species (ROS), malondialdehyde (MDA), and protein carbonyls and downregulated NRF2/HO-1, was significantly counteracted by MYR-LNPs. In addition, MYR-LNPs mitigated MTX-evoked inflammation and nitrosative stress by reducing *NF-κB*, *TNF-α*, *IL-1β*, nitric oxide, and *iNOS* expression. They corrected apoptotic imbalance by lowering Bax and caspase 3 while increasing Bcl-2. Histopathological and ultrastructural assessments confirmed that MYR-LNPs preserved hepatic architecture and mitochondrial integrity. These findings indicate that MYR-loaded liposomal nanoantioxidants provide superior protection against MTX-induced hepatotoxicity by modulating oxidative stress, inflammation, and apoptosis, supporting their potential as an advanced nanodrug delivery strategy for antioxidant therapy.

## 1. Introduction

Drug-induced liver injury (DILI) remains a major challenge in contemporary therapeutics, frequently leading to the failure of otherwise effective compounds in clinical trials, post-marketing withdrawal, or the imposition of strict safety restrictions [1,2]. As the central organ for xenobiotic biotransformation, the liver is particularly exposed to reactive metabolites and toxic intermediates that can trigger oxidative injury [3]. The spectrum of hepatotoxicity ranges from transient elevations of serum transaminases to extensive hepatocellular necrosis, inflammation, fibrosis, and in severe cases acute liver failure [4].

Methotrexate (MTX) is a cornerstone agent in oncology and an established steroid-sparing drug for autoimmune disorders such as psoriasis and rheumatoid arthritis [5,6]. Its antifolate, antimetabolite action is mediated by inhibition of dihydrofolate reductase [7]. However, long-term and frequent MTX administration is strongly associated with hepatotoxicity, often necessitating dose reduction or discontinuation and thereby compromising therapeutic efficacy [5]. Although the full molecular cascade underlying MTX-induced liver injury has not been completely delineated, converging evidence implicates oxidative stress as a central pathogenic driver [8]. MTX has been shown to disrupt mitochondrial function, impair the electron transport chain, and enhance reactive oxygen species (ROS) generation [9], leading to DNA damage, lipid peroxidation, and protein oxidation [10]. In parallel, MTX suppresses endogenous antioxidant defenses, including superoxide dismutase (SOD), catalase (CAT), and glutathione peroxidase (GPx), further aggravating redox imbalance [11]. Inflammatory signaling and caspase 3–mediated apoptosis act in concert with oxidative damage to amplify hepatocellular injury [12].

Consequently, strategies that strengthen endogenous antioxidant systems, attenuate oxidative and inflammatory cascades, and modulate apoptosis are attractive for limiting MTX-related hepatotoxicity. Among such approaches, bioactive natural products with potent antioxidant properties have gained considerable attention as hepatoprotective candidates. Myricetin (MYR), a dietary flavonol abundant in grapes, peanuts, berries, and various medicinal plants, exhibits a broad pharmacological profile [13]. MYR can directly scavenge ROS, upregulate cellular antioxidant defenses, prevent lipid peroxidation, and modulate inflammatory and apoptotic pathways. Additionally, it has demonstrated anticancer, antiangiogenic, antiproliferative, and anti-invasive effects in diverse experimental models [14]. These attributes support the hypothesis that MYR may mitigate MTX-induced oxidative liver damage.

Nevertheless, the clinical translation of MYR is hampered by its poor aqueous solubility, chemical instability, limited oral bioavailability, and rapid metabolism, which restrict effective concentrations at target tissues and diminish its in vivo hepatoprotective efficacy [15]. In this context, nanotechnology-based delivery systems have emerged as promising tools to overcome the biopharmaceutical limitations of natural antioxidants. Liposomal nanoparticles are regarded as safe and versatile carriers that can incorporate hydrophobic compounds, thereby improving their apparent solubility, shielding them from premature degradation, and enhancing pharmacokinetics, tissue distribution, and cellular uptake [16,17]. Encapsulating MYR into liposomal nanoparticles (MYR-LNPs) is therefore expected to increase its stability, bioavailability, and hepatic tissue accumulation [18].

Despite growing evidence supporting the hepatoprotective potential of flavonoids, most studies have evaluated these compounds in their free forms, which suffer from poor aqueous solubility, rapid metabolism, and limited hepatic bioavailability, factors that collectively constrain their in vivo efficacy [19,20]. While liposomal and polymeric nanoformulations of structurally related flavonoids, such as quercetin, naringenin, and naringin, have demonstrated enhanced hepatoprotective outcomes compared with their free counterparts in various liver injury models [21,22,23], no study has yet examined the specific potential of myricetin encapsulated within a liposomal nanoantioxidant system in the context of MTX-induced hepatotoxicity. This gap represents the primary motivation and central novelty of the present work.

Accordingly, the present study was designed, to the best of our knowledge, as the first to evaluate a myricetin-loaded liposomal nanoantioxidant formulation in a methotrexate-induced hepatotoxicity model in rats, explicitly comparing it with free myricetin and comprehensively assessing oxidative stress, inflammatory, and apoptotic pathways as mechanistic targets.

## 2. Materials and Methods

### 2.1. Drugs

Methotrexate (MTX; 50 mg/5 mL injectable solution) was obtained from EIPICO (Cairo, Egypt). Myricetin (MYR) was purchased from Sigma-Aldrich (Cairo, Egypt) and freshly dissolved in dimethyl sulfoxide (DMSO) immediately before use. Myricetin-loaded liposomal nanoparticles (MYR-LNPs) were prepared according to the thin-film hydration protocol described below. The MTX dose was selected based on the hepatotoxicity model reported by [24]. At the same time, the MYR dose was adopted from previous work by Sun et al. to ensure biological relevance and comparability [25].

### 2.2. Preparation of Myricetin-Loaded Liposomal Nanoparticles (MYR-LNPs)

**Figure 1:**
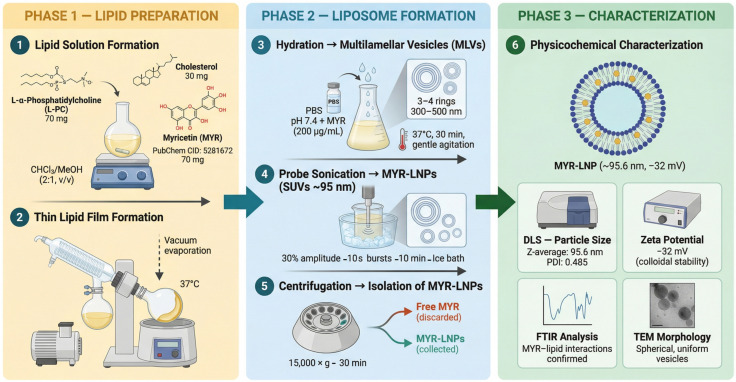
Schematic representation of the stepwise preparation and physicochemical characterization of myricetin-loaded liposomal nanoantioxidants (MYR-LNPs) by the thin-film hydration method. Phase 1 (amber): l-α-phosphatidylcholine (L-PC, 70 mg), cholesterol (30 mg), and myricetin (MYR) are dissolved in chloroform–methanol (2:1, *v*/*v*), followed by vacuum rotary evaporation to yield a thin lipid film. Phase 2 (blue): The film is hydrated with PBS (pH 7.4) containing MYR (200 μg/mL) at 37 °C for 30 min, generating multilamellar vesicles (MLVs), which are subsequently reduced to small unilamellar vesicles (SUVs) by probe sonication (Sonics Vibra-Cell; 30% amplitude, 10 s bursts, 10 min, ice bath); free unencapsulated MYR is removed by centrifugation (15,000× *g*, 30 min). Phase 3 (green): The isolated MYR-LNPs are characterized by dynamic light scattering (DLS), zeta-potential measurement, Fourier-transform infrared spectroscopy (FTIR), and transmission electron microscopy (TEM). Adapted from the open source GAABSTRACT (https://gaabstract.com/) (accessed 28 March 2026).

Myricetin (MYR, ≥98%), cholesterol, and l-α-phosphatidylcholine (L-PC) were sourced from Sigma-Aldrich (Egypt). To prepare the liposomes, L-PC (70 mg; 0.09 mmol) was combined with cholesterol (30 mg; 0.078 mmol), yielding a uniform hydrated lipid mixture at 10 mg/mL. MYR-LNPs were then fabricated using the thin-film hydration technique, as depicted in Figure 1. In this step, accurately measured amounts of cholesterol and L-PC were completely dissolved in 10 mL of a chloroform–methanol mixture (2:1, *v*/*v*) in a round-bottomed flask, yielding a clear, uniform lipid solution ready for subsequent processing. Once the organic solvents were completely evaporated under vacuum, a thin, uniform lipid film remained. This film was then gently hydrated with 5 mL of phosphate-buffered saline (pH 7.4) containing 1 mg of MYR (≈5 μmol), yielding a smooth, well-dispersed formulation with a final drug concentration of 200 μg/mL, ensuring thorough incorporation of MYR throughout the lipid matrix. This technique allows MYR to be mainly incorporated within the lipid bilayer, minimizing its distribution in the aqueous core. Once hydrated, the lipid–drug mixture was incubated at 37 °C with gentle agitation for 30 min, ensuring complete swelling of the lipid layers and promoting maximal drug encapsulation. To obtain smaller, more uniform liposomal particles, the multilayered vesicle suspension was treated with probe sonication using a Sonics Vibra-Cell device (Sonics & Materials, Inc., Newtown, CT, USA), applying 10 s bursts at 30% amplitude intermittently for a total of 10 min. The entire process was carried out in an ice bath to preserve liposome structural integrity and prevent heat accumulation. Free unencapsulated MYR was then separated by centrifugation at 15,000× *g* for 30 min. The resulting supernatant containing the MYR-LNPs was characterized for its physicochemical properties, including particle size, surface charge, and polydispersity index, using a Zetasizer Nano ZS (Malvern Panalytical Ltd., Malvern, UK). Particle size measurements (Z-average) were performed in distilled water, and zeta potential was determined in triplicate to ensure accuracy. The morphology and structural integrity of the liposomal vesicles were assessed using high-resolution transmission electron microscopy (TEM; JEOL 2100, Tokyo, Japan) operating at 160 kV, providing detailed insights into their shape and uniformity.

### 2.3. Encapsulation Efficiency and Drug Loading of MYR

The encapsulation efficiency (EE%) and drug loading (DL%) of MYR in the liposomes were determined indirectly by quantifying free, non-entrapped MYR in the supernatant. After centrifugation, the concentration of unencapsulated MYR was measured using UV-vis spectrophotometry at 260 nm against an appropriate blank. The amount of encapsulated MYR was calculated by subtracting the free drug from the total MYR initially added.

Drug loading and encapsulation efficiency were calculated as:

Drug loading (%) = [Encapsulated MYR/Total liposome mass] × 100

Encapsulation efficiency (%) = [Encapsulated MYR/Total MYR added] × 100

This approach provided a quantitative estimate of the extent of MYR incorporation into the liposomal matrix.

### 2.4. Fourier-Transform Infrared (FTIR) Analysis

Fourier-transform infrared (FTIR) spectroscopy (Bruker, Karlsruhe, Germany) was performed to investigate potential interactions between MYR and the lipid components. Spectra were recorded for pure MYR, L-PC, cholesterol, physical mixtures thereof, and MYR-LNPs using the KBr pellet method over a range of 4000–400 cm^−1^. Characteristic bands of MYR, particularly hydroxyl (–OH) and carbonyl (C=O) stretching vibrations, were examined for shifts or intensity changes after encapsulation, which can indicate physicochemical interactions or complex formation within the liposomal system.

### 2.5. In Vitro Release of Free MYR and MYR-LNPs

The in vitro release profiles of free MYR and MYR-LNPs were evaluated using a dialysis bag diffusion method in PBS (pH 7.4) containing 0.5% Tween 80 to approximate physiological conditions and maintain sink conditions. Aliquots of 2 mL of free MYR solution (200 μg/mL) or MYR-LNP suspension (equivalent to 200 μg/mL MYR) were placed into dialysis bags (molecular weight cutoff 12–14 kDa). Each bag was immersed in 50 mL of release medium maintained at 37 °C and agitated at 100 rpm using a magnetic stirrer.

At predetermined time points (0.5, 1, 2, 4, 8, 12, 24, and 48 h), 2 mL samples were withdrawn from the medium and replaced with an equal volume of fresh, pre-warmed PBS to maintain constant volume and sink conditions. MYR content in the samples was quantified spectrophotometrically at 260 nm. The cumulative percentage of MYR released at each time point was calculated as:

Cumulative release (%) = [Amount of MYR released at time t/Total encapsulated MYR] × 100

### 2.6. Experimental Animals

Sixty adult male albino Sprague Dawley rats (200–300 g) were obtained from the Medical Experimental Research Center (MERC), Faculty of Medicine, Mansoura University (Mansoura, Egypt). On arrival, animals were clinically inspected, then randomly allocated to the experimental groups and housed in standard plastic cages with wood-chip bedding under controlled conditions (25 ± 2 °C, 45 ± 5% relative humidity, 12 h light/dark cycle), with unrestricted access to commercial rodent chow and tap water. A 2-week acclimatization period was allowed before initiating treatments to minimize handling and transport stress and to ensure stable baseline physiological parameters. All procedures involving animals conformed to OECD Test Guideline 420 for acute oral toxicity and were conducted in accordance with internationally accepted principles for the care and use of laboratory animals. The institutional animal ethics committee approved the protocol.

### 2.7. Study Design and Procedures

After acclimatization, rats were randomly allocated into six groups (*n* = 10 per group):

- Group I (control): received vehicle only and no active treatment.
- Group II (MYR): received MYR (50 mg/kg, intraperitoneal [IP]) once daily for 7 consecutive days.
- Group III (MYR-LNPs): received MYR-LNPs (equivalent to 50 mg/kg MYR, IP) once daily for 7 days.
- Group IV (MTX): received a single IP dose of MTX (20 mg/kg) on day 1.
- Group V (MYR + MTX): received MTX (20 mg/kg, IP) on day 1, followed by MYR (50 mg/kg, IP) once daily for 5 days.
- Group VI (MYR-LNPs + MTX): received MTX (20 mg/kg, IP) on day 1, followed by MYR-LNPs (equivalent to 50 mg/kg MYR, IP) once daily for 5 days.

Doses and schedules were chosen based on previous reports and pilot observations to reliably induce hepatotoxicity with MTX [26,27,28] while allowing evaluation of the protective effects of MYR and MYR-LNPs [13,29].

### 2.8. Biological Sampling and Tissue Processing

Before euthanasia, the rats were fasted for 10 h. Anesthesia was induced with isoflurane and maintained until the complete loss of reflexes was confirmed. Subsequently, euthanasia was performed by exposure to a high concentration of isoflurane (5% in oxygen) for 5 min. Cessation of cardiac and respiratory functions was verified to confirm death, with careful attention paid to minimizing animal suffering throughout the procedure. In each group of ten rats, seven were allocated for biochemical and molecular analyses, while the remaining three were reserved for histopathological and ultrastructural evaluations. Following confirmation, blood was withdrawn via the retro-orbital venous plexus and kept at room temperature to allow complete clot formation before subsequent analysis. Serum was obtained by centrifuging the clotted blood at 3000× *g* for 10 min. The clear supernatant was carefully collected, aliquoted, and preserved at −80 °C to maintain sample stability and prevent repeated freeze–thaw cycles.

Immediately after dissection, hepatic tissues were collected and washed with chilled 1.15% KCl solution to remove any remaining traces of blood. Portions of liver tissue designated for biochemical and molecular analyses were stored at −20 °C until use. At the same time, other representative samples were immersed in 10% neutrally buffered formalin for subsequent histological and ultrastructural examinations. For biochemical analyses, liver tissues were gently homogenized in 50 mmol/L Tris–HCl buffer (pH 7.4) to produce a uniform suspension. The homogenates were centrifuged at 10,000× *g* for 15 min at 4 °C. The resulting supernatants were carefully collected and stored at −20 °C to preserve enzymatic activity and molecular integrity until further use.

### 2.9. Biochemical Analysis of Serum Parameters

Serum total protein, albumin, aspartate aminotransferase (AST), alanine aminotransferase (ALT), alkaline phosphatase (ALP), and total bilirubin (TB) were measured using a Hitachi 912 automated biochemical analyzer (Hitachi, Tokyo, Japan) according to the manufacturer’s instructions. Commercial diagnostic kits were supplied by Nanjing Jiancheng Bioengineering Institute (Nanjing, China). Globulin concentrations were calculated as the difference between total protein and albumin. All assays were performed in duplicate, and internal quality controls were included where applicable.

### 2.10. Assessment of Antioxidant Defense and Oxidative Stress

Assessment of hepatic antioxidant defenses and oxidative stress markers was performed in liver homogenates using commercial colorimetric and ELISA kits according to the manufacturers’ instructions. Reduced glutathione (GSH) content and glutathione S-transferase (GST), glutathione peroxidase (GPx), and catalase (CAT) activity were determined with kits from BioDiagnostic (Giza, Egypt). Lipid peroxidation was assessed as malondialdehyde (MDA) using a thiobarbituric acid reactive substances (TBARS) assay, and absorbance values were recorded at the appropriate wavelengths for each assay. Details of all commercial kits and catalog numbers are provided in Supplementary Table S1.

Protein carbonyl (PC) content, an index of protein oxidation, and malondialdehyde-equivalent TBARS levels were measured with rat-specific kits according to the manufacturers’ protocols. Intracellular reactive oxygen species (ROS) generation was evaluated using a DCFH-DA–based fluorescent assay in single-cell suspensions prepared from fresh hepatic tissue, with fluorescence recorded at excitation/emission settings suitable for oxidized DCF and expressed as relative fluorescence units normalized to the control group.

Levels of the redox-sensitive transcription factor nuclear factor erythroid 2–related factor 2 (NRF2) and its downstream effector heme oxygenase 1 (HO-1) were quantified in liver homogenates using sandwich ELISA kits, and concentrations were calculated from standard curves after appropriate dilution. Total protein in tissue extracts was determined and used for normalization where applicable.

### 2.11. Assessment of Key Inflammatory Mediators and Nitrosative Stress

Hepatic levels of key inflammatory mediators were quantified in liver homogenates using rat-specific ELISA kits (MyBioSource, San Diego, CA, USA), including nuclear factor kappa B (NF-κB), tumor necrosis factor α (TNF-α), and interleukin 1β (IL-1β), according to the manufacturers’ protocols. Details of kit catalog numbers are provided in Supplementary Table S1. Each sample was run in triplicate, and cytokine concentrations were calculated from the corresponding standard curves.

Nitrosative stress was evaluated by measuring total nitric oxide (NO) metabolites (nitrate + nitrite) in hepatic tissue using a microplate-based colorimetric assay (MyBioSource, San Diego, CA, USA) according to the manufacturer’s instructions. Absorbance was recorded at the specified wavelength, and NO concentrations were derived from a standard calibration curve and expressed per milligram of tissue protein.

### 2.12. RNA Extraction and cDNA Synthesis

Total RNA was extracted from liver samples using a phenol–guanidinium-based lysis reagent (QIAzol, Qiagen, Hilden, Germany), followed by phase separation with chloroform and alcohol precipitation, according to the manufacturer’s protocol. The RNA pellets were washed with 75% ethanol, briefly air-dried, and dissolved in DNase/RNase-free water. RNA concentration and purity were determined spectrophotometrically at 260, 280, and 230 nm. Only preparations with acceptable 260/280 and 260/230 ratios were used for downstream analysis.

First-strand cDNA was synthesized from 1 μg of total RNA using a reverse-transcription kit (iScript, Bio-Rad, Hercules, CA, USA) in a 20 μL reaction volume following the supplier’s instructions. The reverse-transcription program included incubation at a constant temperature for cDNA synthesis followed by enzyme inactivation, as recommended by the kit manufacturer. Details of the qPCR reagents, reaction components, and instrument specifications are provided in Supplementary Table S2.

### 2.13. Quantitative Real-Time PCR

Quantitative real-time PCR was carried out on a Rotor-Gene Q instrument (Qiagen, Germany) using SYBR Green–based master mix (iTaq Universal SYBR Green Supermix, Bio-Rad, USA). Each 20 μL reaction contained cDNA template, SYBR Green mix, gene-specific primers, and nuclease-free water. Primers targeting oxidative stress–related, inflammatory, and apoptotic genes, as well as the housekeeping gene β-actin, were synthesized commercially (Macrogen, Seoul, Republic of Korea), and their sequences and expected amplicon sizes are listed in Table 1. Before its selection as the reference gene, the expression stability of β-actin was evaluated alongside multiple candidate housekeeping genes across all experimental groups. β-actin consistently demonstrated the most stable expression with minimal intergroup variability, and was therefore selected as the internal reference gene for normalization of all relative mRNA expression data [30].

Thermal cycling consisted of an initial denaturation step, followed by 40 amplification cycles with gene-specific annealing temperatures and a final melting-curve analysis to verify amplification specificity. Relative mRNA expression levels were calculated using the 2^–ΔΔCt^ method [31] with β-actin as the internal reference gene, and all samples were run in triplicate, including no-template controls to exclude contamination.

### 2.14. Apoptotic Marker Profiling

Hepatic levels of the pro-apoptotic protein Bax, the anti-apoptotic protein Bcl-2, and the executioner caspase 3 were quantified in liver homogenates using rat-specific ELISA kits (MyBioSource, San Diego, CA, USA) following the manufacturer’s protocols. Details of all ELISA kits and catalog numbers used for apoptotic and MAPK markers are summarized in Table S1. Bax, Bcl-2, and caspase 3 concentrations were measured from standard curves and normalized to total protein content where appropriate, and all samples were analyzed in duplicate to ensure assay reliability.

### 2.15. Quantification of Phosphorylated MAPK Signaling Proteins

The phosphorylated forms of ERK1/2, p38 MAPK, and JNK in hepatic tissue were determined using SimpleStep ELISA kits (Abcam, Cambridge, UK) according to the manufacturer’s instructions (Table S1). Before ELISA, total protein in liver homogenate supernatants was measured using the bicinchoninic acid (BCA) method and used to normalize phosphorylated protein levels. Samples were assayed in duplicate, and absorbance was read at 450 nm. Data are expressed as phosphoprotein content relative to total protein to reflect activation of these MAPK pathways.

### 2.16. Liver Histopathology

Tissue samples intended for histopathological analysis were first immersed in 10% formalin at a 20:1 fixative-to-tissue ratio and left for three days to ensure proper fixation. Afterward, the samples underwent a gradual dehydration process using an ascending series of alcohols, followed by clearing in xylene. After clearing, the tissues were placed in molten paraffin for one hour to ensure complete infiltration, then carefully embedded in paraffin blocks. Thin sections were cut using a rotary microtome, passed through graded alcohols for dehydration, and finally stained with hematoxylin and eosin to allow detailed histological examination. Histological sections were carefully prepared and observed under a light microscope. The extent of hepatic steatosis was then assessed using a semi-quantitative approach according to the established AASLD criteria (Table 2) to provide a clear, standardized evaluation of liver changes. Comprehensive histopathological assessments were performed on three rats per group. For each slide, three liver sections were included, and every section was thoroughly examined across four distinct microscopic fields at 400× magnification, resulting in a total of 12 fields analyzed per animal. Lesion severity was assessed using a semi-quantitative scale from 0 (no detectable lesion) to 3 (severe), and an average score per rat was determined across all evaluated microscopic fields.

### 2.17. Transmission Electron Microscopy (TEM)

For ultrastructural examination, liver specimens were fixed in 2.5% glutaraldehyde in 0.1 M phosphate buffer (pH 7.4) at 4 °C, followed by post-fixation in 1% osmium tetroxide, dehydration through graded ethanol series and acetone, and embedding in epoxy resin following standard TEM protocols. Ultrathin sections (60–70 nm) were cut with an ultramicrotome, mounted on copper grids, and stained sequentially with uranyl acetate and lead citrate to enhance contrast. Ultrastructural features of hepatic cells and mitochondria were assessed using a JEOL JEM-2100 transmission electron microscope (JEOL Ltd., Tokyo, Japan) operated at 160 kV [32].

### 2.18. Immunohistochemical Assay of NRF2 and NF-κB

Paraffin-embedded hepatic sections were deparaffinized, rehydrated through graded alcohols, and subjected to heat-induced antigen retrieval in citrate buffer (pH 6.0). Endogenous peroxidase activity was blocked with hydrogen peroxide, and non-specific binding was minimized by preincubation with bovine serum albumin. Sections were then incubated overnight at 4 °C with rabbit antibodies against NRF2 and NF-κB p65 (Abcam, Cambridge, UK; details of clones, dilutions, and catalogue numbers are provided in Supplementary Table S1), followed by a biotinylated secondary antibody and streptavidin–horseradish peroxidase complex.

Immunoreactivity was visualized using 3,3′-diaminobenzidine as the chromogen and counterstained with hematoxylin before dehydration and mounting. Stained sections were examined under a light microscope at high power, and digital images were captured from multiple non-overlapping fields per section. The proportions of positively stained areas for NRF2 and NF-κB were quantified using ImageJ (version 1.54) and expressed as a percentage of the total tissue area analyzed.

### 2.19. Statistical Analysis

Data were analyzed using SAS software (version 2012; SAS Institute Inc., Cary, NC, USA). A sample size of 10 rats per group was used, based on previous studies in similar preclinical models, to ensure sufficient power to detect treatment effects [33,34,35]. The distribution of variables and equality of variances were examined before applying parametric tests. All datasets met parametric assumptions, with normality confirmed by the Shapiro–Wilk test and homogeneity of variance verified using Levene’s test. Group differences were assessed by one-way analysis of variance (ANOVA), followed when appropriate by Tukey’s post hoc test for multiple comparisons. For variables that did not satisfy normality or variance assumptions, nonparametric alternatives or log transformation were applied as appropriate. Results are expressed as means ± standard error (SE), and differences were considered statistically significant at *p* < 0.05. Graphs were generated using GraphPad Prism 9.0 (GraphPad Software, San Diego, CA, USA).

## 3. Results

### 3.1. Physicochemical Characterization of MYR-LNPs

**Figure 2:**
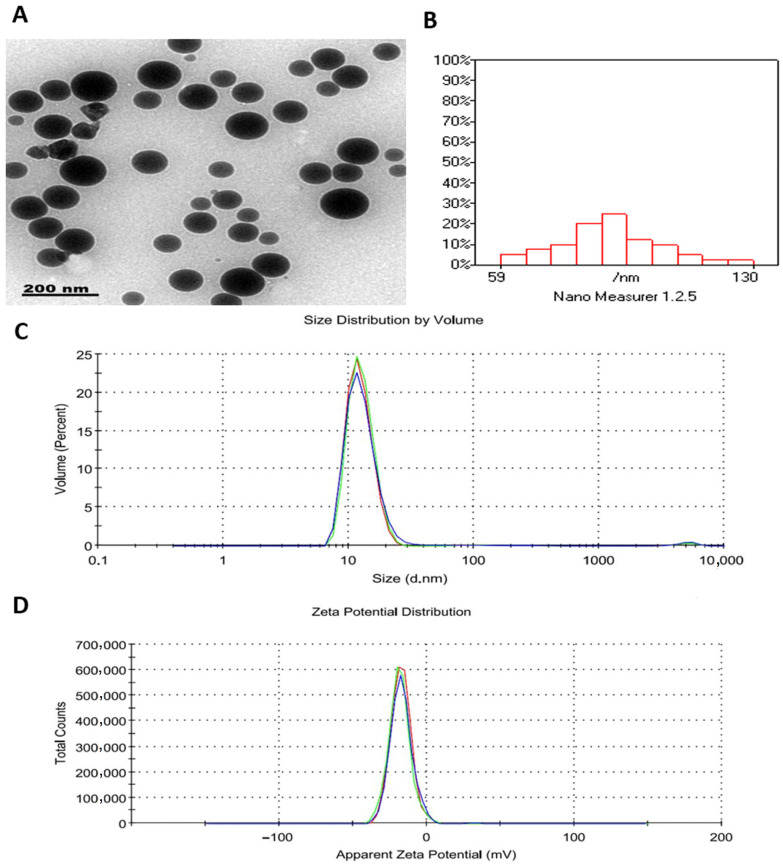
Physicochemical characterization of myricetin-loaded liposomal nanoantioxidants (MYR-LNPs). (**A**) TEM micrograph showing predominantly spherical, well-dispersed vesicles without obvious aggregation. (**B**) Particle size distribution histogram indicating that most particles lay between 60 and 130 nm. (**C**) DLS profile illustrating a mean hydrodynamic diameter of approximately 95 nm and a narrow size distribution. (**D**) Zeta-potential distribution demonstrating a negative surface charge around −32 mV, consistent with good colloidal stability of the nanosystem.

Transmission electron microscopy showed that MYR-LNPs were predominantly spherical, with smooth contours and uniform dispersion, and no obvious aggregation was observed (Figure 2A). Particle size analysis indicated that most vesicles fell within 60–130 nm, reflecting a relatively narrow size range (Figure 2B). Dynamic light scattering revealed a mean hydrodynamic diameter of 95.6 nm with a polydispersity index (PDI) of 0.485, confirming a moderately homogeneous nanosized population (Figure 2C). The zeta potential of the formulation was −32 mV (Figure 2D), indicating a negatively charged surface that supports electrostatic repulsion and suggests acceptable colloidal stability under the tested conditions. In addition, the formulation maintained comparable particle size and zeta-potential values during the short-term observation period, with reproducible measurements obtained across independently prepared batches, indicating acceptable temporal stability and batch repeatability. However, serum stability was not evaluated in the present study and warrants further investigation.

### 3.2. Encapsulation Efficiency and Drug Loading of MYR

The MYR-loaded liposomal nanoparticles exhibited an encapsulation efficiency of 87%, indicating that the majority of the added myricetin was successfully incorporated into the lipid bilayer. The drug loading was calculated to be 13%, reflecting effective incorporation relative to the total lipid mass.

### 3.3. Fourier-Transform Infrared (FTIR) Analysis

**Figure 3:**
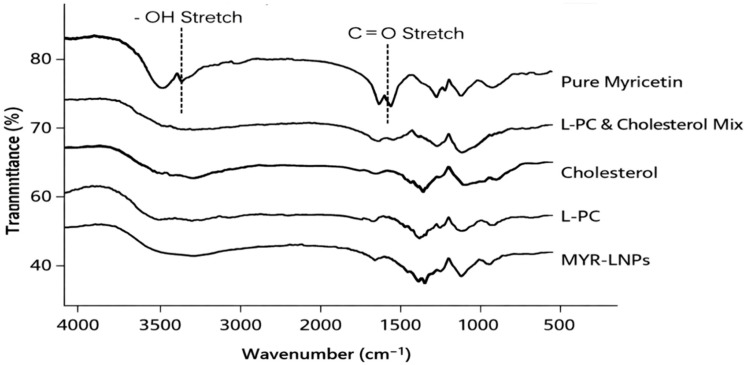
FTIR spectra of myricetin, lipid components, physical mixture thereof, and myricetin-loaded liposomal nanoantioxidants (MYR-LNPs). Spectra highlight the characteristic hydroxyl (–OH) and carbonyl (C=O) bands of myricetin and show slight shifts and intensity changes after encapsulation, indicating interactions between myricetin and the lipid matrix while preserving the drug’s functional groups.

FTIR spectra supported successful incorporation of MYR into the liposomal matrix (Figure 3). Pure MYR showed characteristic absorption bands attributed to hydroxyl (–OH) and carbonyl (C=O) functional groups, while L-PC and cholesterol exhibited their typical phospholipid and sterol signatures. In the spectrum of MYR-LNPs, the principal MYR bands were still detectable, but appeared slightly shifted and with altered intensities compared with free MYR, suggesting intermolecular interactions between MYR and the lipid constituents. The physical mixture spectrum largely represented a superposition of the individual components, whereas the MYR-LNP spectrum showed a more integrated pattern, consistent with MYR being embedded within the lipid bilayer. These findings indicate that the thin-film hydration method produced a stable liposomal system with successful MYR encapsulation.

### 3.4. In Vitro Release of Myricetin

**Figure 4:**
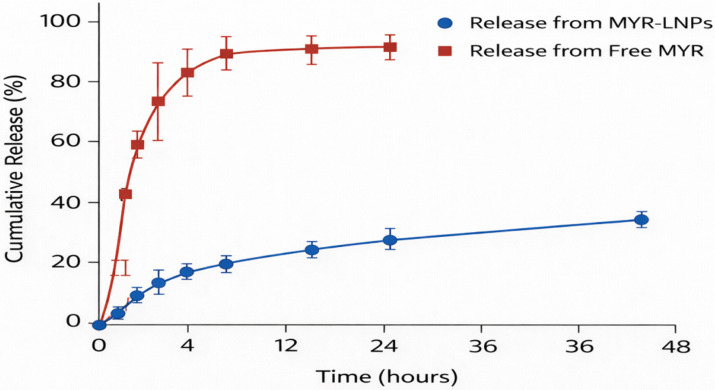
In vitro release profiles of free myricetin (MYR) and myricetin-loaded liposomal nanoantioxidants (MYR-LNPs) in PBS (pH 7.4) containing Tween 80 at 37 °C. Free MYR exhibits rapid release, approaching complete diffusion within 24 h. In contrast, MYR-LNPs show a slower, sustained release over 48 h, reflecting controlled drug liberation from the liposomal bilayer and prolonged nanoantioxidant availability.

Free MYR exhibited a rapid release pattern, with approximately 40% of the dose diffusing through the dialysis membrane within 30 min, around 60% by 1 h, and nearly complete release (~100%) by 24 h (Figure 4). In contrast, MYR-LNPs released MYR in a slower, sustained fashion. About 15% of the drug was released within the first 30 min, increasing to roughly 55% at 8 h and reaching approximately 85% by 48 h. This sustained-release profile is consistent with MYR being largely associated with the liposomal bilayer rather than freely dispersed in the aqueous phase. These findings indicate prolonged release behavior that may contribute to improved formulation performance; however, direct pharmacokinetic confirmation of prolonged in vivo availability and stability was not performed in the present study.

### 3.5. Effect of MYR-LNPs on MTX-Induced Liver Dysfunction

**Figure 5:**
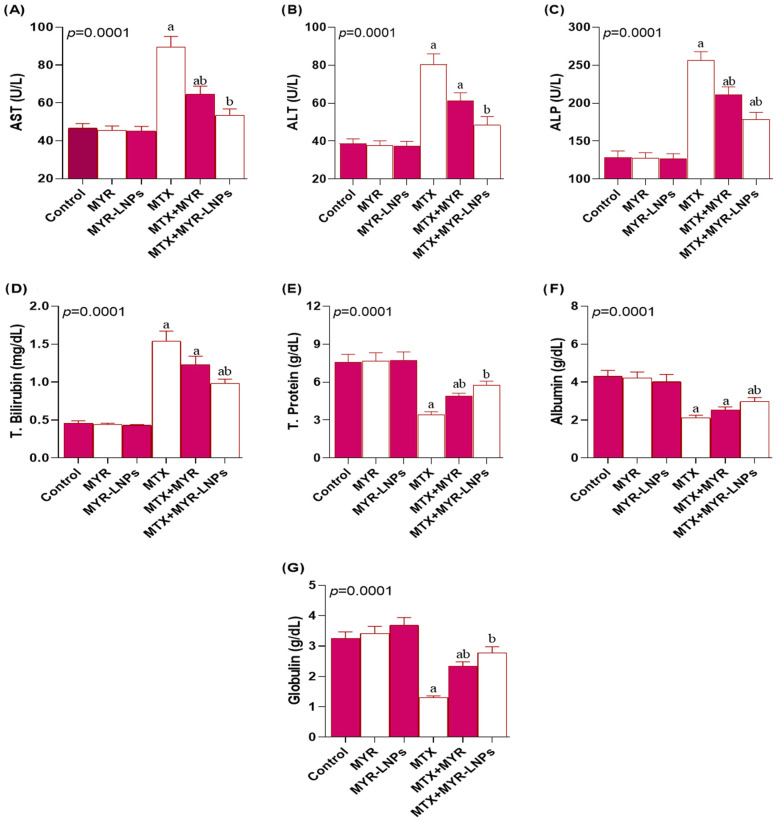
Effect of myricetin and myricetin-loaded liposomal nanoantioxidants (MYR-LNPs) on serum liver function markers in methotrexate (MTX)-induced hepatotoxicity. Serum (**A**) AST, (**B**) ALT, (**C**) ALP, (**D**) total bilirubin, (**E**) total protein, (**F**) albumin, and (**G**) globulin levels in control and treated groups. MYR-LNPs more effectively normalized MTX-induced alterations than free myricetin, indicating superior preservation of hepatocellular integrity and synthetic function. Data are presented as means ± SD, and statistical significance was considered at *p* < 0.05. Values annotated with superscript “a” denote a statistically significant difference relative to the normal control group, whereas those labeled “b” indicate a statistically significant difference compared with the MTX-treated group.

MTX administration significantly impaired liver function, as indicated by increased serum AST, ALT, and ALP activities compared with the control group (Figure 5A–C). Co-treatment with MYR-LNPs significantly attenuated these elevations and produced greater reductions than crude MYR. Notably, AST and ALT activities in the MTX + MYR-LNP group were restored to values that did not differ from those of the control animals.

Serum total bilirubin was also significantly higher in MTX-treated rats than in controls (Figure 5D). MYR-LNP co-treatment significantly lowered bilirubin levels, whereas crude MYR did not produce a significant change relative to the MTX group. Conversely, MTX administration significantly decreased serum total protein, albumin, and globulin compared with controls (Figure 5E–G). These reductions were significantly reversed by MYR-LNPs, with improvements that exceeded those observed with free MYR.

### 3.6. Effect of MYR-LNPs on Antioxidant Status and Oxidative Stress

**Figure 6:**
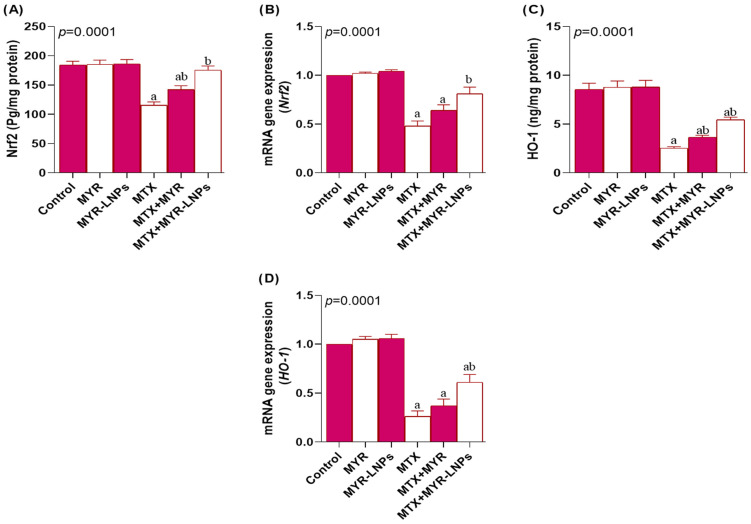
Modulation of the NRF2–HO-1 antioxidant axis by myricetin and myricetin-loaded liposomal nanoantioxidants (MYR-LNPs) in methotrexate (MTX)-treated rats. Protein and mRNA levels of (**A**,**B**) NRF2 and (**C**,**D**) HO-1. MTX suppressed NRF2/HO-1, while MYR-LNPs, more than free MYR, restored this pathway toward control levels, consistent with enhanced activation of endogenous antioxidant defenses by the nanoantioxidant formulation. Data are presented as means ± SD, and statistical significance was considered at *p* < 0.05. Values annotated with superscript “a” denote a statistically significant difference relative to the normal control group, whereas those labeled “b” indicate a statistically significant difference relative to the MTX-treated group.

MTX disrupted the NRF2–HO-1 antioxidant axis, leading to a significant decline in both NRF2 and HO-1 at the protein and mRNA levels compared with the control group (Figure 6A–D). MYR-LNP co-treatment significantly restored NRF2 and HO-1 expression to levels comparable with those of control rats, whereas crude MYR did not significantly affect NRF2 or HO-1 transcripts in MTX-treated animals.

**Figure 7:**
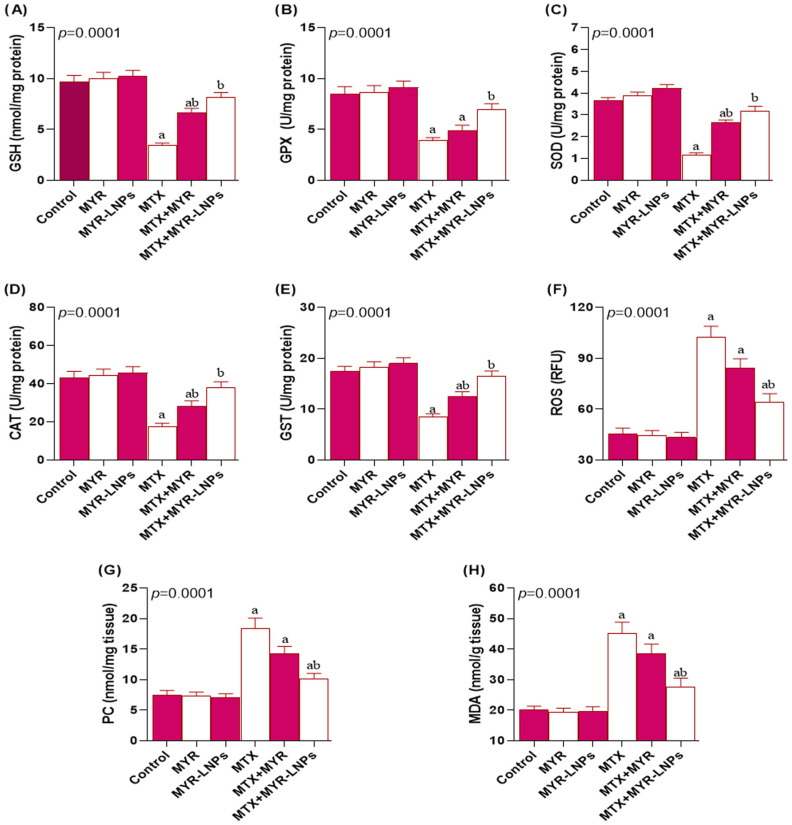
Effect of myricetin and myricetin-loaded liposomal nanoantioxidants (MYR-LNPs) on hepatic antioxidant status and oxidative stress in methotrexate (MTX)-induced hepatotoxicity. Hepatic (**A**) GSH, (**B**) GPx, (**C**) SOD, (**D**) CAT, (**E**) GST, and (**F**–**H**) oxidative markers (ROS, MDA, protein carbonyls). MTX depleted antioxidant defenses and increased oxidative damage. In contrast, MYR-LNPs produced a more complete restoration of enzymatic and non-enzymatic antioxidants and a greater reduction in ROS, lipid peroxidation, and protein oxidation than free MYR. Data are presented as means ± SD, and statistical significance was considered at *p* < 0.05. Values annotated with superscript “a” denote a statistically significant difference relative to the normal control group, whereas those labeled “b” indicate a statistically significant difference compared with the MTX-treated group.

Consistent with these findings, hepatic GSH content and the activities of GPx, SOD, CAT, and GST were significantly reduced in the MTX group compared with controls (Figure 7A–E). Administration of MYR-LNPs significantly normalized these antioxidant parameters, and values in the MTX + MYR-LNP group no longer differed from controls. By contrast, crude MYR did not significantly improve GPx activity compared with MTX alone.

Oxidative stress markers were markedly increased after MTX exposure. Hepatic ROS, MDA, and protein carbonyl levels were all significantly higher than in the control group (Figure 7F–H). Co-treatment with MYR-LNPs significantly reduced these indices of oxidative damage and was consistently more effective than crude MYR, which did not significantly differ from the MTX group for several oxidative parameters.

### 3.7. Effect of MYR-LNPs on Inflammatory and Nitrosative Markers

**Figure 8:**
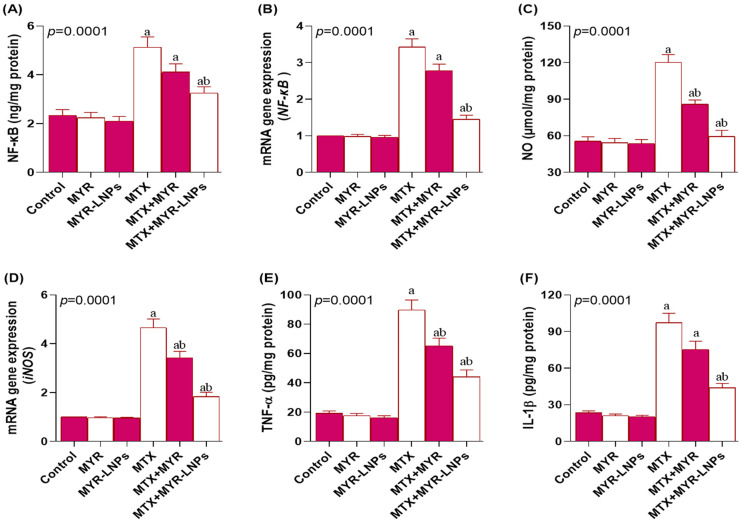
Impact of myricetin and myricetin-loaded liposomal nanoantioxidants (MYR-LNPs) on inflammatory and nitrosative mediators in methotrexate (MTX)-treated rats. (**A**,**B**) NF-κB protein and mRNA, (**C**) hepatic NO, (**D**) iNOS mRNA, and (**E**,**F**) serum TNF-α and IL-1β. MTX markedly activated NF-κB and nitrosative/pro-inflammatory pathways. MYR-LNPs reduced NF-κB activation, NO/iNOS, and pro-inflammatory cytokines more effectively than free MYR, indicating stronger nanoantioxidant-mediated anti-inflammatory effects. Data are presented as means ± SD, and statistical significance was considered at *p* < 0.05. Values annotated with superscript “a” denote a statistically significant difference relative to the normal control group, whereas those labeled “b” indicate a statistically significant difference relative to the MTX-treated group.

MTX markedly activated inflammatory signaling, as evidenced by significant increases in NF-κB protein and gene expression levels compared with controls (*p* < 0.05; Figure 8A,B). MYR-LNPs significantly suppressed NF-κB activation, whereas crude MYR failed to significantly alter NF-κB protein or mRNA levels relative to MTX alone.

MTX also enhanced nitrosative stress. Hepatic NO levels and iNOS mRNA expression were significantly elevated in MTX-treated rats versus controls (Figure 8C,D). Both MYR and MYR-LNPs significantly decreased NO and iNOS, with the nanoliposomal formulation producing the more pronounced reduction.

In addition, MTX significantly raised serum concentrations of the pro-inflammatory cytokines TNF-α and IL-1β (Figure 8E,F). Co-administration of MYR-LNPs significantly lowered both cytokines compared with MTX alone. Crude MYR also reduced TNF-α, but did not significantly change IL-1β relative to the MTX group.

### 3.8. Effect of MYR-LNPs on MAP Signaling

**Figure 9:**
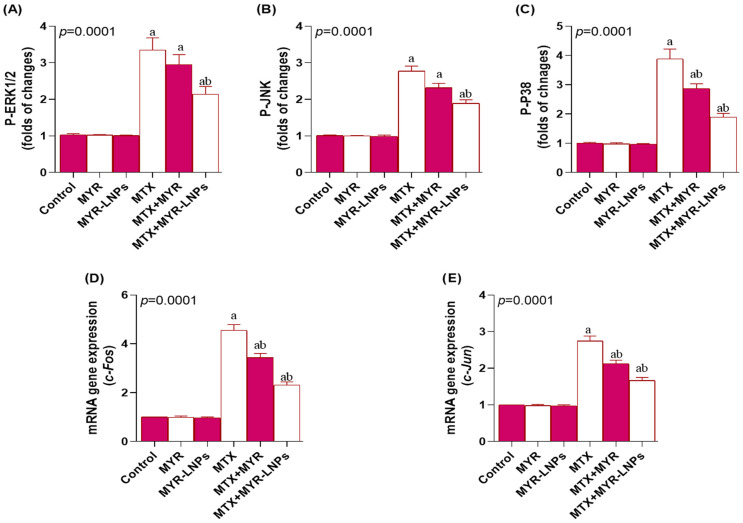
Effect of myricetin and myricetin-loaded liposomal nanoantioxidants (MYR-LNPs) on MAPK signaling in methotrexate (MTX)-induced hepatotoxicity. Hepatic levels of (**A**) p-ERK1/2, (**B**) p-JNK, (**C**) p-p38, and downstream transcription factors (**D**) c-Fos and (**E**) c-Jun. MTX robustly activated MAPK/AP-1 signaling, whereas MYR-LNPs, more than free MYR, significantly downregulated these phosphorylated kinases and transcription factors, suggesting efficient suppression of stress- and inflammation-related MAPK pathways by the nanoantioxidant formulation. Data are presented as means ± SD, and statistical significance was considered at *p* < 0.05. Values annotated with superscript “a” denote a statistically significant difference relative to the normal control group, whereas those labeled “b” indicate a statistically significant difference compared with the MTX-treated group.

Activation of the MAPK pathway was evident in MTX-treated rats, which exhibited significantly elevated hepatic levels of p-ERK1/2, p-JNK, p-p38, c-Fos, and c-Jun compared with controls (Figure 9A–E). MYR-LNP co-treatment significantly downregulated these phosphorylated MAPKs and their downstream transcription factors, indicating attenuation of MTX-induced MAPK activation. Free MYR exerted a weaker effect, and p-ERK1/2 and p-JNK levels in the MTX + MYR group did not differ significantly from those in the MTX group.

### 3.9. Effect of MYR-LNPs on Apoptotic Markers

**Figure 10:**
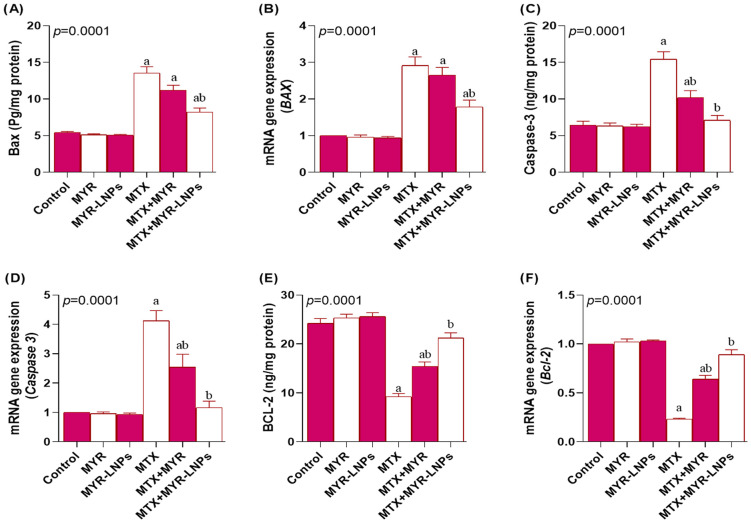
Modulation of apoptotic markers by myricetin and myricetin-loaded liposomal nanoantioxidants (MYR-LNPs) in methotrexate (MTX)-treated rat livers. Hepatic protein and mRNA levels of (**A**,**B**) Bax, (**C**,**D**) caspase 3, and (**E**,**F**) Bcl-2. MTX increased pro-apoptotic Bax and caspase 3 and decreased anti-apoptotic Bcl-2, whereas MYR-LNPs more effectively than free MYR normalized these markers and restored Bcl-2, indicating stronger protection against mitochondrial apoptosis by the nanoantioxidant delivery system. Data are presented as means ± SD, and statistical significance was considered at *p* < 0.05. Values annotated with superscript “a” denote a statistically significant difference relative to the normal control group, whereas those labeled “b” indicate a statistically significant difference compared with the MTX-treated group.

MTX exposure significantly enhanced hepatic apoptosis, as reflected by increased Bax and caspase 3 at both the protein and transcript levels compared with controls (Figure 10A–D). MYR-LNP treatment significantly reduced Bax and caspase 3 levels, with caspase 3 levels in the MTX + MYR-LNP group returning to values not significantly different from those in the control group. Free MYR produced only partial protection; Bax protein and mRNA in the MTX + MYR group remained comparable to MTX alone.

In contrast, the anti-apoptotic protein Bcl-2 was significantly decreased by MTX at both the protein and gene expression levels (Figure 10E,F). Co-treatment with either MYR or MYR-LNPs significantly increased Bcl-2 expression, with a more marked effect in the MYR-LNP group, where Bcl-2 values were restored to control levels (adjusted *p* > 0.05 vs. control, Tukey’s post hoc test).

### 3.10. Histopathological Findings

**Figure 11:**
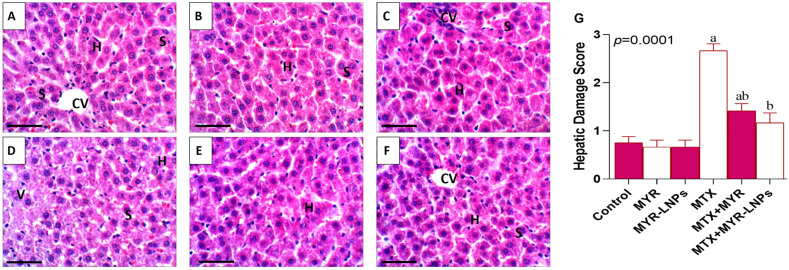
Representative photomicrographs of liver sections obtained from the control and different experimental groups: (**A**) Control group; (**B**) MYR-treated group; (**C**) MYR-LNP-treated group; (**D**) MTX-treated group; (**E**) MTX + MYR group; and (**F**) MTX + MYR-LNP group. CV, central vein; S, hepatic sinusoids; H, hepatocytes; V, cytoplasmic vacuolation. All images were captured at ×400 magnification (scale bar = 50 µm). (**G**) Semi-quantitative histopathological scores of liver damage in control and treated groups. Data are presented as means ± SD, and statistical significance was considered at *p* < 0.05. Values annotated with superscript “a” denote a statistically significant difference relative to the normal control group, whereas those labeled “b” indicate a statistically significant difference compared with the MTX-treated group.

H&E-stained liver sections from the control, MYR, and MYR-LNP groups showed preserved hepatic architecture, including normal central veins, radiating hepatic cords, and hepatocytes with polygonal morphology, eosinophilic cytoplasm, and centrally located nuclei (Figure 11A–C). In contrast, MTX-treated livers displayed marked pathological changes, characterized by hepatocellular vacuolation, central vein congestion, and nuclear necrosis, indicative of pronounced structural damage (Figure 11D).

Rats co-treated with MYR or MYR-LNPs exhibited substantial attenuation of these lesions. Liver architecture was largely restored, with hepatocytes regaining near-normal arrangement and morphology, particularly in the MYR-LNP group (Figure 11E,F).

Semi-quantitative histological scores were significantly higher in MTX-exposed rats than in controls (Figure 11G). Both MYR and MYR-LNPs significantly reduced these scores, with the nanoliposomal formulation conferring the greatest improvement in overall liver histology.

### 3.11. Ultrastructural Findings

**Figure 12:**
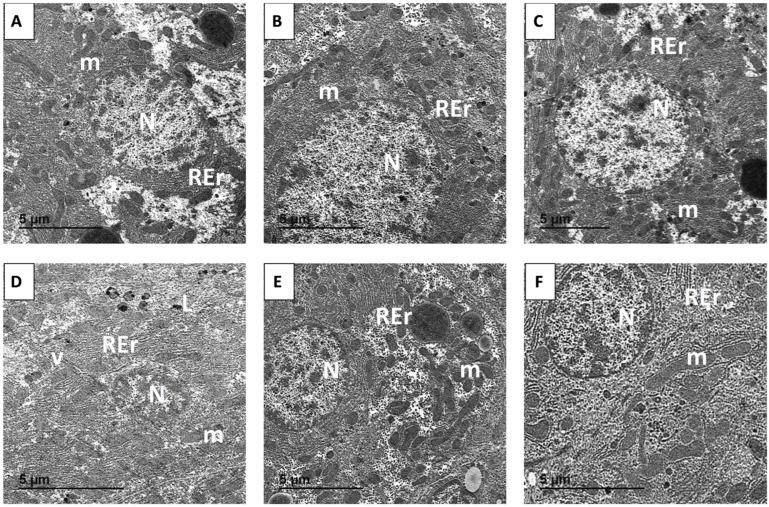
Representative transmission electron micrographs illustrating the hepatic ultrastructure in the control and different experimental groups: (**A**) Control group; (**B**) MYR-treated group; (**C**) MYR-LNP-treated group; (**D**) MTX-treated group; (**E**) MTX + MYR group; and (**F**) MTX + MYR-LNP group. N, nucleus; m, mitochondria; REr, rough endoplasmic reticulum; v, cytoplasmic vacuolation; L, lysosomes.

EM analysis revealed normal ultrastructure in hepatocytes from the control, MYR, and MYR-LNP groups. Cells displayed intact plasma membranes, centrally located nuclei with prominent nucleoli, well-organized rough endoplasmic reticulum, and mitochondria with intact membranes and clearly defined cristae. Bile canaliculi and sinusoidal spaces appeared normal (Figure 12A–C).

In MTX-treated rats, hepatocytes showed severe ultrastructural disruption, including cytoplasmic vacuolization, marked swelling and dilatation of rough endoplasmic reticulum, mitochondrial swelling with distorted or lost cristae, chromatin condensation, and nuclear irregularities (Figure 12D). Co-treatment with MYR or MYR-LNPs markedly mitigated these changes. Hepatocytes from these groups exhibited largely restored mitochondrial morphology, improved endoplasmic reticulum organization, and nuclei with more regular contours, consistent with substantial protection against MTX-induced subcellular damage (Figure 12E,F).

### 3.12. NRF2 Immunohistochemistry

**Figure 13:**
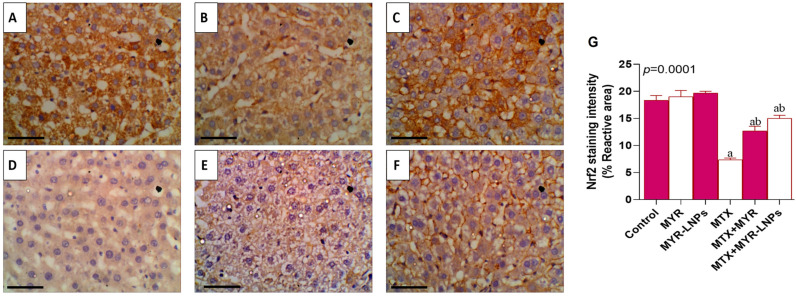
Representative photomicrographs illustrating NRF2 immunolocalization in rat liver tissue. (**A**–**C**) Liver sections from the control groups exhibited strong positive NRF2 immunoreactivity within hepatocytes. (**D**) The MTX-treated group showed mild NRF2 immunostaining in hepatocytes. (**E**,**F**) In the treated groups, NRF2 immunoreactivity was markedly increased, demonstrating strong positive staining within hepatocytes. Images were captured at an original magnification of ×400 (scale bar = 50 µm). (**G**) Quantitative analysis of NRF2 immunostaining intensity in rat liver across experimental groups; Data are presented as means ± SD, and statistical significance was considered at *p* < 0.05. Values annotated with superscript “a” denote a statistically significant difference relative to the normal control group, whereas those labeled “b” indicate a statistically significant difference compared with the MTX-treated group.

In the control, MYR, and MYR-LNP groups, NRF2 immunostaining was strong and widespread within hepatocytes, reflecting robust basal NRF2 expression (Figure 13A–C). MTX treatment markedly reduced NRF2 immunoreactivity, which appeared weak and focal in hepatocytes (Figure 13D). MYR, and more prominently MYR-LNP, co-administration restored NRF2 staining intensity toward control levels (Figure 13E,F).

Quantitative image analysis confirmed a significant decrease in NRF2-positive area in the MTX group compared with controls, and a significant increase in both MYR- and MYR-LNP-treated groups, with the highest values in the MYR-LNP group (*p* < 0.05; Figure 13G).

### 3.13. NF-κB Immunohistochemistry

**Figure 14:**
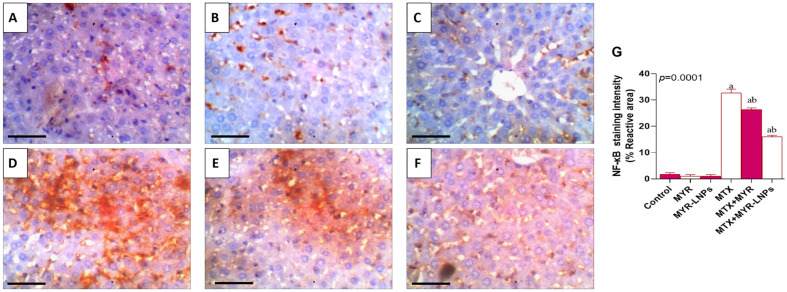
Representative photomicrographs illustrating NF-κB protein immunoreactivity in rat liver tissue. (**A**–**C**) Liver sections from the control groups showed no detectable NF-κB immunostaining. (**D**) The MTX-treated group demonstrated strong NF-κB immunolocalization in hepatocytes, evidenced by intense brown staining, indicating hepatic injury. (**E**,**F**) The treated groups exhibited partial inhibition of NF-κB expression, as reflected by weak immunostaining that was comparable to that of the normal control group. Images were captured at an original magnification of ×400 (scale bar = 50 µm). (**G**) Quantitative analysis of NRF2 immunostaining intensity in rat liver across experimental groups; Data are presented as means ± SD, and statistical significance was considered at *p* < 0.05. Values annotated with superscript “a” denote a statistically significant difference relative to the normal control group, whereas those labeled “b” indicate a statistically significant difference compared with the MTX-treated group.

Control, MYR, and MYR-LNP livers showed minimal or absent NF-κB immunostaining (Figure 14A–C). In contrast, MTX-treated liver sections showed intense NF-κB immunoreactivity in hepatocytes, consistent with marked NF-κB activation (Figure 14D). Treatment with MYR or MYR-LNPs substantially reduced NF-κB staining, which appeared weak and approached control levels, with the lowest immunoreactivity observed in the MYR-LNP group (Figure 14E,F).

Quantitative analysis demonstrated a significant increase in NF-κB–positive area in the MTX group versus controls and a significant reduction in both MYR- and MYR-LNP-treated groups (Figure 14G).

## 4. Discussion

This study demonstrates that myricetin delivered as liposomal nanoantioxidants (MYR-LNPs) provides substantially greater protection against MTX-induced hepatotoxicity than free myricetin, underscoring the value of nanocarriers for optimizing natural antioxidant therapy. MTX caused a characteristic pattern of liver injury, characterized by significant increases in ALT, AST, ALP, and total bilirubin, accompanied by reductions in total protein and albumin, indicating compromised hepatocellular integrity and synthetic capacity [36]. These findings are consistent with previous reports that MTX hepatotoxicity is closely linked to excessive ROS generation, depletion of cellular reducing equivalents, mitochondrial dysfunction, and weakened endogenous antioxidant defenses [8,28]. Elevated bilirubin suggests impaired hepatic uptake, conjugation, or excretion, whereas reduced albumin reflects disturbed protein synthesis, which is highly sensitive to oxidative and inflammatory stress [37,38].

Co-administration of MYR-LNPs markedly corrected these biochemical disturbances, normalizing transaminases and improving protein indices more effectively than free MYR. These data are in line with earlier work showing that MYR preserves hepatocellular function by dampening NF-κB–driven inflammation and enhancing Nrf2-dependent antioxidant responses [39]. The superior effect of MYR-LNPs likely reflects improved bioavailability, prolonged circulation, and preferential hepatic accumulation, all recognized advantages of nanoliposomal delivery in liver-targeted therapy [40]. In this context, the zeta potential of −32 mV recorded for MYR-LNPs is particularly noteworthy. This level of surface electronegativity confers robust electrostatic repulsion between adjacent vesicles, preventing aggregation and ensuring long-term colloidal stability during circulation, consistent with the well-established threshold of ±30 mV required for adequate electrostatic stabilization of liposomal formulations [41,42,43]. Moreover, the negative surface charge facilitates favorable interactions with the fenestrated sinusoidal endothelium of the liver, promoting selective hepatic uptake. Negatively charged liposomes are internalized up to 10-fold more efficiently than neutral counterparts by liver sinusoidal endothelial cells via scavenger receptor–mediated endocytosis [44]. This charge-selective hepatotropism is further supported by recent evidence showing that nanosized carriers approximately 100 nm in size are preferentially and rapidly sequestered by liver sinusoidal endothelial cells due to their unique sinusoidal location and high clearance capacity [45]. Collectively, these physicochemical attributes position MYR-LNPs as a rationally designed hepatotropic carrier capable of delivering therapeutically relevant myricetin concentrations directly to the site of MTX-induced injury.

Oxidative and nitrosative stress emerged as central events in MTX-induced liver damage. MTX significantly increased hepatic MDA and protein carbonyls, indicating extensive lipid peroxidation and protein oxidative modification, in agreement with the established oxidative profile of MTX injury [46,47]. Concurrently, NO levels and iNOS expression were elevated, favoring peroxynitrite formation and amplifying damage to lipids, proteins, and DNA [48]. These changes were accompanied by a marked depletion in GSH and reduced activities of SOD, CAT, GPx, and GST, reflecting a collapse of enzymatic and non-enzymatic antioxidant defenses. Collectively, these alterations indicate that MTX shifts the redox balance toward a strongly pro-oxidant state, consistent with the broader literature on ROS-driven liver injury [49,50,51].

Free MYR partially reversed these oxidative insults by lowering MDA, PC, and NO while restoring GSH and antioxidant enzyme activities, in keeping with its known role as a direct radical scavenger and Nrf2 activator [14,39]. Importantly, MYR-LNPs produced a more profound normalization of both antioxidant defenses and oxidative/nitrosative markers and restored NRF2/HO-1 expression to near-control levels. This suggests that nanoencapsulation enhances MYR’s ability to activate the NRF2 axis and reinforce downstream antioxidant pathways [52,53]. As illustrated in Figure 6, MYR-LNPs produced a statistically significant upregulation of both NRF2 and HO-1 protein and mRNA expression compared with the MTX group and the free MYR group, providing direct quantitative evidence that the liposomal formulation amplifies the canonical cytoprotective NRF2/HO-1 response in hepatic tissue. It should be noted, however, that these mechanistic conclusions are inferred from convergent biochemical, molecular, and immunohistochemical evidence rather than directly demonstrated through functional experiments such as pathway-specific inhibitor studies, NRF2 nuclear translocation assays, or siRNA-mediated knockdown approaches. Future studies employing such tools will be valuable to formally establish the causal roles of the NRF2/HO-1, NF-κB, and MAPK pathways in mediating the hepatoprotective effects of MYR-LNPs and to delineate the precise molecular hierarchy governing their interactions. Our findings resonate with those of Eki Nci-Akdemi et al., who showed that MYR attenuates MTX-induced oxidative stress by reducing lipid peroxidation and restoring SOD, CAT, and GPx activities [24]. Here, the liposomal formulation further advances this benefit, consistent with recent evidence that nanoliposomes can markedly improve the pharmacokinetic and pharmacodynamic profiles of antioxidant compounds [54,55].

Inflammation is a major downstream consequence of oxidative stress in the liver and is tightly regulated by NF-κB and MAPK pathways [56,57]. In this study, MTX markedly upregulated NF-κB at both protein and transcript levels and increased TNF-α, IL-1β, NO, and iNOS, indicating robust inflammatory and nitrosative responses. In parallel, there was pronounced activation of MAPK signaling, as shown by elevated p-ERK1/2, p-JNK, and p-p38 and their downstream AP-1 components c-Fos and c-Jun. Together, these changes highlight cross talk between redox imbalance, MAPK activation, and NF-κB–dependent cytokine production [58,59]. Quantitative data presented in Figure 9 demonstrate that MYR-LNPs significantly suppressed the phosphorylation of ERK1/2, JNK, and p38, as well as the downstream AP-1 components c-Fos and c-Jun, to a greater extent than free MYR, directly linking the superior anti-inflammatory outcome of the nanoformulation to its more effective attenuation of MAPK cascade activation.

Free MYR attenuated several of these inflammatory mediators, in line with prior studies showing that MYR suppresses NF-κB activation, reduces pro-inflammatory cytokine expression, and modulates macrophage polarization [60]. MYR-LNPs, however, produced more consistent downregulation of NF-κB, MAPKs, TNF-α, IL-1β, and iNOS. This enhanced anti-inflammatory effect is likely attributable to sustained intracellular MYR exposure and more effective modulation of upstream oxidative stress, which in turn dampens NF-κB and MAPK signaling [61].

Apoptosis represents a key downstream event linking oxidative stress and inflammation with hepatocyte loss. In agreement with previous reports on MTX toxicity [62], MTX elevated Bax and caspase 3 and reduced Bcl-2, shifting the Bax–Bcl-2 balance toward mitochondrial apoptosis. Free MYR partially corrected this imbalance, restoring Bcl-2 and lowering caspase 3, consistent with its reported anti-apoptotic properties in liver injury models [60]. Notably, MYR-LNPs more effectively normalized Bax, caspase 3, and Bcl-2 and restored caspase 3 to control levels, indicating superior protection of mitochondrial integrity and apoptotic control. These anti-apoptotic effects likely stem from the combined attenuation of oxidative stress, NF-κB, and MAPK activation achieved with the nanoformulation [61,63].

Histopathological and ultrastructural findings provided strong morphological support for the biochemical and molecular data. MTX caused extensive architectural disruption, hepatocellular degeneration, inflammatory infiltration, vacuolization, and pronounced mitochondrial damage, all consistent with ROS-driven and inflammatory liver injury. These structural changes mirror the elevated transaminases, high bilirubin, depleted antioxidant status, and activated apoptotic markers observed in MTX-treated rats. In contrast, MYR-LNPs preserved the classical lobular architecture, reduced inflammatory infiltrates, and maintained near-normal hepatocyte and mitochondrial ultrastructure. The close agreement between structural preservation, restoration of liver function tests, enhancement of NRF2/HO-1, suppression of NF-κB/MAPK signaling, and normalization of apoptosis strongly supports a multi-target hepatoprotective mechanism for MYR-LNPs [29].

From a broader perspective, our data fit within the emerging paradigm that nanocarrier-mediated delivery is an effective strategy to overcome the poor stability and bioavailability of natural antioxidants and to achieve meaningful hepatoprotection [64,65,66]. By integrating a well-characterized nanoformulation with comprehensive redox, inflammatory, apoptotic, and histological endpoints, this work provides mechanistic in vivo evidence that nanoantioxidants can meaningfully improve the therapeutic performance of flavonoids, such as myricetin, in drug-induced liver injury. While our study demonstrates the superiority of liposomal encapsulation in enhancing myricetin’s hepatoprotective efficacy, it is important to situate these findings within the broader context of nanoformulation strategies for drug-induced liver injury. Polymeric nanoparticles, particularly PLGA- and PEG–PCL-based systems, have been shown to enhance the hepatoprotective activity of natural polyphenols such as curcumin and resveratrol by improving their solubility, sustained release, and hepatic accumulation compared with free forms [67]. Similarly, chitosan-based magnetic nanoparticles loaded with curcumin have demonstrated hepatoprotection against chemically induced liver toxicity in rats through synergistic antioxidant and anti-inflammatory mechanisms [68]. More recently, oxidation-responsive PEGylated nanoparticles encapsulating CoQ10 significantly attenuated hepatic ischemia–reperfusion injury by targeting ROS scavenging and ferroptosis [69]. Notably, nanoliposomal delivery of naringin, structurally related to myricetin as a flavonoid, was shown to outperform free naringin in restoring hepatic antioxidant defenses and suppressing inflammatory and apoptotic signaling in a nickel-induced hepatotoxicity model [22]. Collectively, these findings reinforce that nanoencapsulation, regardless of the carrier platform, consistently enhances the hepatic bioavailability and efficacy of natural antioxidants, and that liposomal systems offer additional advantages in terms of biocompatibility and surface tunability for hepatic targeting [67,68,69,70].

## 5. Study Limitations and Future Directions

Despite the comprehensive mechanistic evidence provided, several limitations of the present study warrant acknowledgment. First, while the simultaneous and consistent modulation of the NRF2/HO-1, NF-κB, and MAPK pathways, along with their downstream effectors, constitutes robust convergent evidence of pathway engagement, formal mechanistic causality was not established through pathway-specific inhibitor studies, siRNA-mediated knockdown, or hepatocyte-specific overexpression models. Such approaches represent an important and necessary extension of the current findings and are prioritized as future research directions. Additionally, the exclusively in vivo design of the present study, while maximizing physiological and translational relevance, precludes direct cellular-level mechanistic interrogation. Complementary in vitro studies employing isolated primary hepatocytes, HepG2, or HepaRG cell models treated with MTX and MYR-LNPs would provide valuable mechanistic resolution, enabling direct assessment of nanoparticle uptake kinetics, intracellular ROS dynamics, and pathway-specific activation of NRF2/HO-1, NF-κB, and MAPK signaling at the hepatocellular level, thereby reducing dependence on whole-animal interpretations and strengthening the mechanistic conclusions of the present work.

Second, the claim of superior hepatic bioavailability of MYR-LNPs over free myricetin, while strongly supported by the physicochemical profile of the formulation, specifically the mean particle size of 95.6 nm and the zeta potential of −32 mV, both of which are well-established determinants of hepatic accumulation, and by the consistently superior pharmacodynamic outcomes across all measured endpoints, should be interpreted cautiously, as direct pharmacokinetic, tissue distribution, or ADME studies were not performed in the present work. Therefore, enhanced bioavailability or hepatic targeting cannot be conclusively confirmed. The improved efficacy observed in the MYR-LNP group compared with free myricetin suggests improved formulation performance and delivery efficiency, but this remains indirect evidence rather than formal pharmacokinetic confirmation. Dedicated studies incorporating plasma concentration-time profiling and fluorescence- or radiolabel-based tissue distribution imaging are therefore warranted to provide a rigorous quantitative pharmacokinetic basis for these functional advantages. Additionally, although the in vitro release study demonstrated sustained MYR release from the nanoparticles, formal kinetic modeling (e.g., Higuchi or Korsmeyer–Peppas) and comprehensive stability assessments, including temporal stability and batch repeatability, were not conducted, which limits mechanistic interpretation and predictive in vivo correlation.

Third, the present study was conducted exclusively in a short-term male rat model, and the long-term in vivo safety, biocompatibility, and immunogenicity of the liposomal carrier itself remain to be evaluated. Sex-based differences in drug metabolism and hepatic response, as well as the performance of MYR-LNPs in chronic MTX exposure models, should be addressed in future investigations. Additionally, an empty liposome control group was not included, limiting the ability to distinguish the potential effects of the carrier from those of the encapsulated MYR. Furthermore, clinical application, dosing feasibility, and potential toxicity of MYR-LNPs in humans remain unknown and require dedicated preclinical safety studies and carefully designed clinical trials.

Furthermore, the present study did not include a conventional hepatoprotective reference drug comparator, such as N-acetylcysteine or silymarin, which would have provided a clinically meaningful pharmacological benchmark for the efficacy of MYR-LNPs. Future studies incorporating such comparators, alongside MYR-LNPs, free MYR, and standard-of-care hepatoprotective agents, will be essential to establish the relative therapeutic advantage of the nanoformulation definitively and to more rigorously assess its translational and clinical potential in the context of MTX-induced liver injury. Ultimately, well-designed preclinical studies in larger animal models and subsequently carefully controlled clinical trials will be essential to establish the safety, optimal dosing regimen, and therapeutic window of MYR-LNPs in patients receiving methotrexate-based treatment regimens.

## 6. Conclusions

MTX administration in rats induced severe hepatotoxicity characterized by impaired liver function, redox imbalance, activation of inflammatory and nitrosative pathways, and enhanced mitochondrial apoptosis. Free myricetin attenuated several of these disturbances, but its efficacy was clearly limited. In contrast, myricetin-loaded liposomal nanoantioxidants provided markedly superior protection: they normalized liver enzymes and protein indices, restored NRF2/HO-1 and major antioxidant defenses, dampened NF-κB/MAPK-driven inflammation and nitrosative stress, and rebalanced Bax/Bcl-2 and caspase 3. Histological and ultrastructural analyses confirmed that MYR-LNPs preserved hepatic architecture and organelle integrity far more effectively than free MYR.

Taken together, these findings identify MYR-LNPs as a promising nanoantioxidant strategy to prevent or mitigate MTX-induced hepatotoxicity and support the broader concept that nanotechnology-enabled delivery can unlock the full hepatoprotective potential of natural antioxidants.

## Figures and Tables

**Table 1:** Gene-specific primers used for quantitative RT-PCR.

Gene	Sequences (5′–3′)	Accession No.	Length (bp)
*Nrf2*	F: TTTGTAGATGACCATGAGTC R: TCCTGCCAAACTTGCTCCAT	NM_031789.2	161
*HO-1*	F: ATGTCCCAGGATTTGTCCGA R: ATGGTACAAGGAGGCCATCA	NM_012580.2	144
*NF* *-* *κB*	F: AGTCCCGCCCCTTCTAAAAC R: CAATGGCCTCTGTGTAGCCC	NM_001276711.1	105
*iNOS*	F: CAGCTGGGCTGTACAAACCT R: CATTGGAAGTGAAGCGTTTC	NM_012611.3	120
*Caspase 3*	F: ACTGGAATGTCAGCTCGCAA R: GCAGTAGTCGCCTCTGAAGA	NM_012922.2	270
*Bax*	F: TTTCATCCAGGATCGAGCAG R: AATCATCCTCTGCAGCTCCA	NM_017059.2	154
*Bcl2*	F: GACTTTGCAGAGATGTCCAG R: TCAGGTACTCAGTCATCCAC	NM_016993.2	214
*c-Fos*	F: CCCGTAGACCTAGGGAGGAC R: CAATACACTCCATGCGGTTG	NM_022197.3	181
*c-Jun*	F: CCAACCAACGTGAGTGCAAG R: CGTCCCCGCTTCAGTAACAA	NM_031203.2	115
*β-Actin*	F: CAGCCTTCCTTCTTGGGTATG R: AGCTCAGTAACAGTCCGCCT	NM_031144.3	360

Nrf2, nuclear factor erythroid 2–related factor 2; HO-1, heme oxygenase 1; NF κB, nuclear factor kappa B; iNOS, inducible nitric oxide synthase; caspase 3, cysteine–aspartic acid protease 3; Bax, Bcl-2–associated X protein; Bcl2, B-cell lymphoma 2; c-Fos, FBJ murine osteosarcoma viral oncogene homolog; c Jun, Jun proto-oncogene; β-actin (beta-actin, housekeeping).

**Table 2:** Semi-quantitative scoring system for liver histopathological changes.

Score	Liver Composite Assessment Score
0 (none)	No pathological changes
1 (mild)	Mild, scattered hepatocellular degeneration and necrosis, minimal inflammatory cell infiltration, occasional vascular congestion
2 (moderate)	Multifocal hepatocellular necrosis, mild vascular congestion, moderate vacuolar degeneration, and pronounced inflammatory cell infiltration
3 (severe)	Extensive hepatocyte necrosis, marked hepatocellular degeneration, moderate to severe vascular congestion, and dense inflammatory cell infiltration

## Data Availability

The original contributions presented in this study are included in the article/Supplementary Material. Further inquiries can be directed to the corresponding author.

## Supplementary Materials

The following supporting information can be downloaded at https://www.mdpi.com/article/10.3390/antiox15040452/s1. Table S1: Commercial kits used for biochemical, oxidative stress, inflammatory, apoptotic, and MAPK signaling assays. Table S2: Quantitative real-time reverse-transcription polymerase chain reaction (qRT-PCR) reagents and instrument specifications.

## Abbreviations

The following abbreviations are used in this manuscript.

ALP	alkaline phosphatase
ALT	alanine aminotransferase
ANOVA	analysis of variance
AP-1	activator protein 1
AST	aspartate aminotransferase
Bax	Bcl-2–associated X protein
BCA	bicinchoninic acid
Bcl-2	B-cell lymphoma 2
BSA	bovine serum albumin
c-Fos	FBJ murine osteosarcoma viral oncogene homolog
c-Jun	Jun proto-oncogene
CAT	catalase
CDNB	1-chloro-2,4-dinitrobenzene
DAB	3,3′-diaminobenzidine
DCFH-DA	2′,7′-dichlorofluorescin diacetate
DILI	drug-induced liver injury
DL	drug loading
DLS	dynamic light scattering
DMSO	dimethyl sulfoxide
DTNB	5,5′-dithiobis(2-nitrobenzoic acid)
EE	encapsulation efficiency
ERK1/2	extracellular signal-regulated kinases 1/2
FTIR	Fourier-transform infrared
GPx	glutathione peroxidase
GSH	reduced glutathione
GST	glutathione S-transferase
H&E	hematoxylin and eosin
HO-1	heme oxygenase-1
HRP	horseradish peroxidase
HSD	honest significant difference
IL-1β	interleukin 1 beta
iNOS	inducible nitric oxide synthase
IP	intraperitoneal
JNK	c-Jun N-terminal kinase
L-PC	l-α-phosphatidylcholine
MAPK	mitogen-activated protein kinase
MDA	malondialdehyde
MERC	Medical Experimental Research Center
MTX	methotrexate
MYR	myricetin
MYR-LNPs	myricetin-loaded liposomal nanoparticles
NF-κB	nuclear factor kappa B
NO	nitric oxide
NRF2	nuclear factor erythroid 2–related factor 2
OECD	Organization for Economic Cooperation and Development
p38	p38 mitogen-activated protein kinase
PBS	phosphate-buffered saline
PC	protein carbonyl
PDI	polydispersity index
qRT-PCR	quantitative real-time polymerase chain reaction
RFU	relative fluorescence unit
ROS	reactive oxygen species
SE	standard error
SOD	superoxide dismutase
TB	total bilirubin
TBARS	thiobarbituric acid reactive substances
TEM	transmission electron microscopy
TNF-α	tumor necrosis factor alpha
