# Supplementary material for: Liposomal Myricetin Nanoantioxidants Attenuate Methotrexate-Induced Hepatotoxicity by Modulating Oxidative Stress, Inflammation, and Apoptosis in Rats
Source: Antioxidants (Basel). 2026 Apr 4;15(4):452. doi: 10.3390/antiox15040452 (PMC13113062; doi:10.3390/antiox15040452)
Supplement: Supplementary file 1 [file antioxidants-15-00452-s001.zip › antioxidants-4227874-supplementary.pdf]

Supplementary Table S1. Commercial kits used for biochemical, oxidative stress, inflammatory, apoptotic, and MAPK signaling assays.

**Assay/target**	**Analyte type**	**Kit/assay name**	**Supplier (country)**	**Catalog No.**	**Detection principle / readout (brief)**
Reduced glutathione (GSH)	Antioxidant	GSH colorimetric kit	BioDiagnostic (Egypt)	2511	DTNB reaction: yellow chromophore, absorbance at 412 nm
Glutathione S-transferase (GST)	Antioxidant enzyme	GST activity kit	BioDiagnostic (Egypt)	2531	GSH–substrate conjugation, absorbance at 340 nm
Glutathione peroxidase (GPx)	Antioxidant enzyme	GPx activity kit	BioDiagnostic (Egypt)	2524	Coupled NADPH oxidation, absorbance at 340 nm
Catalase (CAT)	Antioxidant enzyme	CAT activity kit	BioDiagnostic (Egypt)	2517	H_2O_2 decomposition, change in absorbance at 510 nm
Malondialdehyde (MDA)	Lipid peroxidation	TBARS/MDA assay	BioDiagnostic (Egypt)	MD 2529	MDA–TBA adduct, absorbance at 532 nm
Protein carbonyl (PC)	Protein oxidation	Rat PC ELISA kit	MyBioSource (USA)	MBS760520	Sandwich ELISA, colorimetric readout
Reactive oxygen species (ROS)	Intracellular ROS	ROS assay kit (DCFH-DA)	MyBioSource (USA)	MBS2540517	DCFH-DA fluorescence, Ex 488 nm / Em 525 nm
8-OhdG (8-hydroxy-2′-deoxyguanosine)	DNA oxidation	Rat 8-OHdG ELISA kit	MyBioSource (USA)	MBS732375	Competitive ELISA, colorimetric
NRF2 (Nuclear factor erythroid 2–related factor 2)	Transcription factor	Rat NRF2 ELISA kit	MyBioSource (USA)	MBS3807961	Sandwich ELISA, colorimetric
HO-1 (Heme oxygenase-1)	Antioxidant enzyme	Rat HO-1 ELISA kit	MyBioSource (USA)	MBS2024438	Sandwich ELISA, colorimetric
NF-κB (nuclear factor kappa B)	Inflammatory transcription factor	Rat NF-κB ELISA kit	MyBioSource (USA)	MBS287521	Sandwich ELISA, colorimetric detection at 450 nm
TNF-α (tumor necrosis factor-α)	Pro-inflammatory cytokine	Rat TNF-α ELISA kit	MyBioSource (USA)	MBS282960	Sandwich ELISA, colorimetric detection at 450 nm
IL-1β (interleukin-1β)	Pro-inflammatory cytokine	Rat IL-1β ELISA kit	MyBioSource (USA)	MBS232385	Sandwich ELISA, colorimetric detection at 450 nm
Total nitric oxide (NOx)	Nitrosative stress marker	Total nitrate/nitrite microplate assay	MyBioSource (USA)	MBS8243214	Griess-based colorimetric assay for NO metabolites

Bax (Bcl-2–associated X protein)	Apoptotic regulator	Rat Bax ELISA kit	MyBioSource (USA)	MBS2512405	Sandwich ELISA<br><br>colorimetric detection at 450 nm
Bcl-2 (B-cell lymphoma-2)	Anti-apoptotic protein	Rat Bcl-2 ELISA kit	MyBioSource (USA)	MBS457882	Sandwich ELISA<br><br>colorimetric detection at 450 nm
Caspase-3	Apoptotic effector protease	Rat Caspase-3 ELISA kit	MyBioSource (USA)	MBS743552	Sandwich ELISA<br><br>colorimetric detection at 450 nm
Phospho-ERK1/2 (Thr202/Tyr204)	MAPK signaling protein	SimpleStep ELISA Kit (phospho-ERK1/2)	Abcam (UK)	ab176660	SimpleStep sandwich ELISA<br><br>colorimetric 450 nm
Phospho-p38 MAPK (Thr180/Tyr182)	MAPK signaling protein	SimpleStep ELISA Kit (phospho-p38)	Abcam (UK)	ab176664	SimpleStep sandwich ELISA<br><br>colorimetric 450 nm
Phospho-JNK (Thr183/Tyr185)	MAPK signaling protein	SimpleStep ELISA Kit (phospho-JNK)	Abcam (UK)	ab176662	SimpleStep sandwich ELISA<br><br>colorimetric 450 nm

Supplementary Table S2. Quantitative Real-Time reverse transcription polymerase chain reaction (qRT-PCR) reagents and instrument specifications.

Reagent/instrument	Category	Commercial name	Supplier (country)	Catalog No.	Notes/application
QIAzol lysis reagent	RNA extraction	QIAzol Lysis Reagent	Qiagen (Germany)	79306	Phenol/guanidinium reagent for total RNA isolation
Reverse transcription kit	cDNA synthesis	iScript cDNA Synthesis Kit	Bio-Rad (USA)	1708891	First-strand cDNA synthesis for qPCR
qPCR master mix	qPCR chemistry	iTaq Universal SYBR Green Supermix	Bio-Rad (USA)	172-5122	SYBR Green-based 2× qPCR master mix
Real-time cycler	Instrument	Rotor-Gene Q real-time PCR cycler	Qiagen (Germany)	–	Real-time PCR instrument used for qRT-PCR
